# Anti-proliferative and anti-secretory effects of everolimus on human pancreatic neuroendocrine tumors primary cultures: is there any benefit from combination with somatostatin analogs?

**DOI:** 10.18632/oncotarget.17008

**Published:** 2017-04-10

**Authors:** Amira Mohamed, David Romano, Alexandru Saveanu, Catherine Roche, Manuela Albertelli, Federica Barbieri, Thierry Brue, Patricia Niccoli, Jean-Robert Delpero, Stephane Garcia, Diego Ferone, Tullio Florio, Vincent Moutardier, Flora Poizat, Anne Barlier, Corinne Gerard

**Affiliations:** ^1^ Aix Marseille Univ, CNRS, CRN2M, Marseille, France; ^2^ APHM, Conception Hospital, Molecular Biology Laboratory, Marseille, France; ^3^ Department of Internal Medicine and Center of Excellence for Biomedical Research, University of Genova, Genova, Italy; ^4^ APHM, Conception Hospital, Endocrinology Department, Marseille, France; ^5^ Paoli Calmettes Cancer Institute, Oncology Department, IPC CoE-ENETS, Marseille, France; ^6^ Paoli Calmettes Cancer Institute, Surgery Department, IPC CoE-ENETS, Marseille, France; ^7^ Paoli Calmettes Cancer Institute, Biopathology Department, IPC CoE-ENETS, Marseille, France; ^8^ APHM, North Hospital, Pathology Laboratory, Marseille, France; ^9^ APHM, North Hospital, Surgery Department, Marseille, France

**Keywords:** everolimus, somatostatin analogs, human pancreatic neuroendocrine tumors, primary culture, co-treatment

## Abstract

Therapeutic management of gastroenteropancreatic neuroendocrine tumors (GEP-NETs) is challenging. The mammalian target of rapamycin (mTOR) inhibitor everolimus recently obtained approval from the Food and Drug Administration for the treatment of patients with advanced pancreatic neuroendocrine tumors (pNETs). Despite its promising antitumor efficacy observed in cell lines, clinical benefit for patients is unsatisfactory. The limited therapeutic potential of everolimus in cancer cells has been attributed to Akt activation due to feedback loops relief following mTOR inhibition. Combined inhibition of Akt might then improve everolimus antitumoral effect. In this regard, the somatostatin analog (SSA) octreotide has been shown to repress the PI3K/Akt pathway in some tumor cell lines. Moreover, SSAs are well tolerated and routinely used to reduce symptoms caused by peptide release in patients carrying functional GEP-NETs. We have recently established and characterized primary cultures of human pNETs and demonstrated the anti-proliferative effects of both octreotide and pasireotide. In this study, we aim at determining the antitumor efficacy of everolimus alone or in combination with the SSAs octreotide and pasireotide in primary cultures of pNETs. Everolimus reduced both Chromogranin A secretion and cell viability and upregulated Akt activity in single treatment. Its anti-proliferative and anti-secretory efficacy was not improved combined with the SSAs. Both SSAs did not overcome everolimus-induced Akt upregulation. Furthermore, caspase-dependent apoptosis induced by SSAs was lost in combined treatments. These molecular events provide the first evidence supporting the lack of marked benefit in patients co-treated with everolimus and SSA.

## INTRODUCTION

Gastroenteropancreatic (GEP) neuroendocrine tumors (NETs) is a rare and heterogeneous group of tumors, with recent increased incidence and prevalence [1]. Pancreatic neuroendocrine tumors (pNETs) constitute a distinct subset of GEP-NETs which behave and respond to systemic treatments differently to other GEP-NETs (see [2] for a review). The majority of pNETs are sporadic but some are associated with inherited genetic syndromes such as multiple endocrine neoplasia type 1 syndrome (MEN1) and tuberus sclerosis. They are classified as functional (10-30%) or non functional (50-80%), based on their specific pancreatic hormone hypersecretion [3]. Treatment options range from curative surgery for localized resectable disease to palliation with medical therapies including somatostatin analogs (SSAs), chemotherapy and targeted treatments for advanced and metastatic pNETs [4]. The SSAs octreotide and lanreotide are currently used to reduce symptoms caused by peptides release in a majority of patients carrying functional GEP-NETs [5]. These effects have been credited to their binding to the somatostatin receptor subtype 2 (SST2). SSAs anti-proliferative role has been investigated in PROMID and CLARINET studies. Both long acting octreotide and lanreotide significantly prolonged progression free survival (PFS) respectively in patients with well-differentiated metastatic midguts GEP-NETs and metastatic pNETs [6, 7].

The PI3K/Akt/mTOR pathway integrates growth factor signals and plays a central role in regulating multiple cellular processes such as cell growth, survival and protein synthesis. Several studies have suggested the involvement of this pathway in pNET tumorigenesis and progression. Whole exome sequencing of pNETs identified inactivating mutations of two major negative regulators of the PI3K/Akt/mTOR pathway, the phosphatase and tensin homolog (PTEN) and the tuberous sclerosis 2 (TSC2), in 14% of tumors [8]. Moreover, Missiaglia *et al*. have shown that PTEN and TSC2 downregulation was associated with shorter PFS and overall survival of pNETs patients [9]. On the contrary, activated levels of Akt and mTOR have been observed in a subset of pNETs but without significant correlation with clinicopathological features [10–12]. Overall, these clues have given the rational for the use of mTOR inhibitors in the treatment of pNETs. Everolimus, a mTORC1 inhibitor, induces anti-proliferative effects in the pNET cell line BON [13]. In the phase III RADIANT 3 trial, everolimus significantly prolonged PFS in patients with progressive advanced pNETs compared with placebo [14]. Thus everolimus obtained approval from the Food and Drug Administration (FDA) and the European Medicines Agency (EMA) for the treatment of patients with advanced pNETs and more recently, for NETs of gastrointestinal or lung origin. However, disease stabilization is observed rather than objective response, which remains low (<5%). The modest antitumor activity of everolimus observed in clinical trials may be due to feedback mechanisms favoring pro-survival signals. The loss of S6kinase-dependent negative feedback loop, involving expression of the insulin receptor substrate 1 (IRS-1), leading to the activation of PI3K/Akt and Ras/MAPKinase has been observed in breast cancer cell lines and human breast and colorectal carcinomas after everolimus treatment [15, 16]. Therefore, combined treatments with drugs targeting PI3K/Akt could improve the anti-proliferative effect of everolimus. In this regard, activation of SST2 has been shown to repress PI3K activity by disrupting the interaction between SST2 and the p85 PI3K regulatory subunit in pituitary and pancreatic adenocarcinoma cell lines [17, 18]. Clinical benefits of combined everolimus with octreotide have been evaluated in pNETs in a phase II trial. Concomitant treatment of everolimus and octreotide increased PFS in comparison with everolimus alone, however objective response rate was lesser in combined treatment [19].

We have recently established and characterized primary cultures of human pNETs and demonstrated the anti-proliferative effects of both octreotide and pasireotide [20]. To assess the relevance of everolimus and co-treatments in pNETs, we have used this suitable state-of-the-art preclinical model to evaluate the anti-proliferative and anti-secretory effects of everolimus in single treatment or in combination with the SSAs octreotide and pasireotide from 25 well characterized tumors. The role of PI3K/Akt/mTOR and the MAPKinase ERK1/2 signaling pathways under these treatments has also been monitored.

## RESULTS

### Molecular characteristics of pNET tissues and pNET primary cultures

Mutations of *MEN1*, *DAXX/ATRX*, *PTEN* or down regulation of their respective proteins have been frequently observed in pNETs ([8, 9]. Loss of DAXX/ATRX expression has recently been associated with poor prognosis and might be used to refine prognostic classification [21]. PTEN mutations have been associated with rapamycin sensitivity in different cell lines [22]. In this context, to better characterize patients’ tumors, we searched for *MEN1* mutations from formalin-fixed paraffin-embedded tissue sections (FFPE) (Table 1). PTEN, DAXX and ATRX expression was performed by using immunohistochemistry (IHC) (Table 1). *MEN1* mutations were frequently observed in 68% of the tumors (17/25). PTEN was always found expressed but with varying levels. Low PTEN expression was observed in 28% of the tumors (7/25). ATRX or DAXX expression was lost in 29% of the tumors (7/24). A similar frequency has been obtained by Singhi *et al*. in a large series of pNETs [21]. We did not observe any correlation between PTEN, MEN1 and DAXX/ATRX alterations (not shown), neither between DAXX/ATRX loss and the initial grade of the tumor (p=0.093). However, DAXX/ATRX was lost in 3 out of the 4 grade 3 tumors present in our series (Table 1).

**Table 1:** Molecular characteristics of human pNET tissues

tumor (number)	Grade	*MEN1* (tumoral mutation)	PTEN IHC	ATRX IHC	DAXX IHC
1	G2	No	1(h)	N	P
2	G1	No	3	P	P
3	G2	exon 10 deleted	3	P	P
4	G1	Exon 4: c.660G>A (p.Trp220*)	2	P	NA
5(a)	G2	Exon 2: c.249_252del (p.Ile85Serfs*33)	2	P	P
6	G3	No	3(h)	P	P
7	G1	Exon 3: c.586G>A (p.Val196Ile)	3	P	P
8	G2	Exon 9: c.1226G>A (p.Cys409Tyr)	3(h)	P	N
9	G2	Exon 2: c.244G>C (p.Asp82His)	2	P	N
10	G2	Exon 5: c.833T>C (p.Met278Thr)	3	P	P
11	G2	Exon 8: c.1151A>G p.Glu384Lys	3	P	P
12	G2	Exon 3: c.456T>G (p.Leu152Phe)	1(h)	P	NA
13	G2	No	3	P	P
14	G2	Exon 3: c.526G>A (p.Ala176Thr)	2	P	P
15	G3	Exon 3: c.487G>A (p.Gly163Arg)Exon 10: c.1741G>A (p.Ala581Thr)	1	NA	N
16	G2	Exon7: c.1034C>T p.Ala345Val	1(h)	P	P
17	G2	No	3	P	P
18	G2	Exon 2: c.38T>C (p.Leu13Pro)Exon 9: c.1318C>T (p.Leu440Phe)	1(h)	NA	NA
19	G1	Exon2: c.322C>T (p.Arg108*)	1(h)	P	P
20(a)	G1	Exon 7c.1049C>T	2(h)	P	N
21	G1	No	3	P	P
22	G1	No	3	P	P
23	G3	Exon 2 c.244G>C (p.Asp82His)Exon 3: c.514G>A (p.Asp172Asn)	3	P	N
24	G3	Exon 3: c.514G>A (p.Asp172Asn)	1(h)	N	P
25	G2	No	2	NA	P

IHC (immunohistochemistry). (a) MEN1 syndrome, mutation found at the germinal level and in the tumor. (h) Heterogeneous labeling. 1-3: low to high labeling level. N (negative labeling). P (positive labeling). NA (not available).

We have previously shown that pNET cells in primary culture maintained their neuroendocrine characteristics. In particular, the majority of cells expressed chromogranin A (CgA) and the SST2 receptor. Moreover we observed that their capacity to secrete CgA was regulated by SSAs [20]. Thus we determined the level of SST2 mRNA expression, CgA secretion and its sensitivity to octreotide in the primary cultures of our series (Table 2). We detected SST2 mRNA expression in all the primary culture analyzed. As previously observed SST2 expression level was higher than the other SST subtypes (not shown). We detected CgA secretion in all investigated primary cultures with variable levels. Moreover, except in one primary culture (tumor 2), CgA secretion was significantly decreased by 1nM octreotide (Table 2).

**Table 2:** Molecular characteristics of pNET primary cultures

Tumor (number)	Grade	SST2R (copies/copies GUSβ)	CgA secretion	
			Control (U/l)	octreotide (%inhibition)
1	G2	2.80	325 ± 9	28 ± 1**
2	G1	4.94	170 ± 5	8 ± 5
3	G2	1.57	159 ± 1	49 ± 10*
4	G1	6.67	3941 ± 13	51 ± 4**
5	G2	ND	ND	ND
6	G3	0.32	10 ± 1	44 ± 7*
7	G1	5.08	137 ± 6	93 ± 1***
8	G2	0.96	49 ± 1	34 ± 11*
9	G2	1.62	40 ± 2	39 ± 2**
10	G2	0.50	488 ± 10	72 ± 1*
11	G2	0.53	21 ± 2	37 ± 6*
12	G2	2.23	514 ± 11	71 ± 1***
13	G2	0.88	870 ± 125	63 ± 2**
14	G2	2.75	569 ± 48	16 ± 2*
15	G3	7.62	284 ± 13	93 ± 2**
16	G2	0.96	691 ± 87	76 ± 10*
17	G2	3.65	93 ± 19	47 ± 6*
18	G2	3.94	66 ± 2	36 ± 4**
19	G1	2.35	735 ± 33	36 ± 15*
20	G1	ND	ND	ND
21	G1	3.23	16 ± 2	49 ± 9*
22	G1	2.67	568 ± 21	85 ± 1***
23	G3	1.11	ND	ND
24	G3	0.50	ND	ND
25	G2	6.92	ND	ND

SST2 receptor mRNA was determined as described in patients and methods by real-time PCR. Cells were treated in the absence or in the presence of 1nM octreotide and CgA secretion was measured as described in patients and methods. (*) p<0.05; (**) p<0.01 (***) p<0.001 versus control conditions.

### Effects of everolimus on cell viability

**Figure 1:**
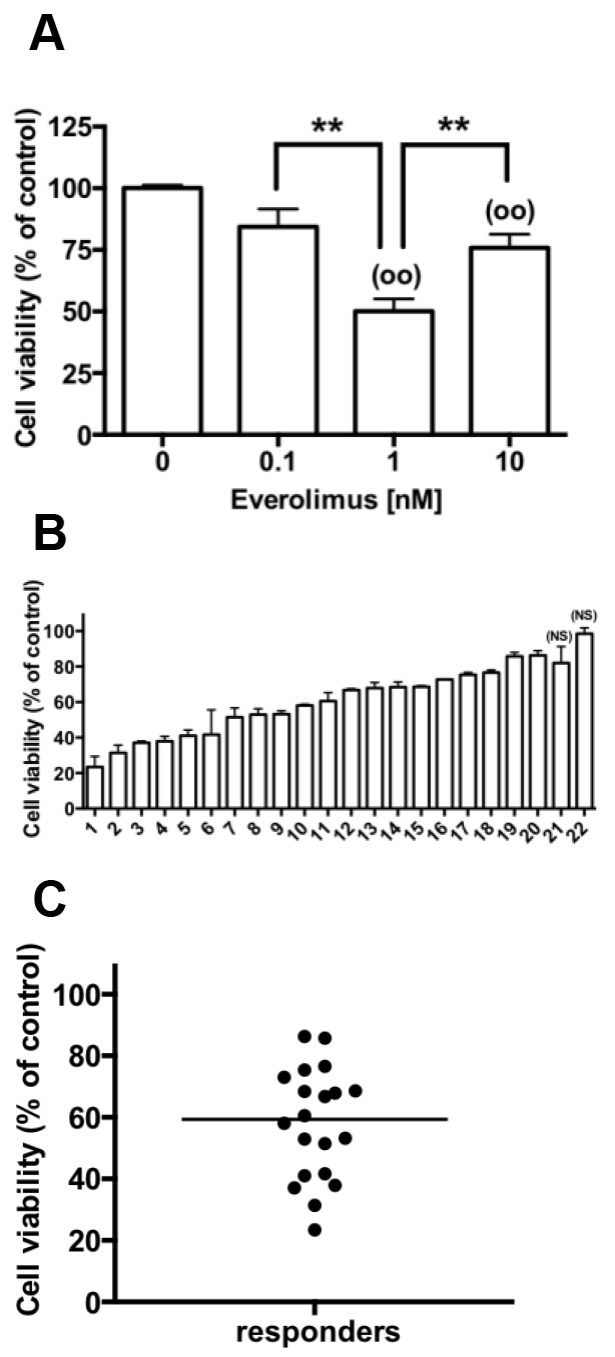
Everolimus decreases cell viability in primary cultures of human pNETs **(A)** Primary culture cells from 3 pNETs (tumors 2,10,11) were incubated without (vehicle with 10^6^ fold DMSO dilution) and with 0.1-10 nM everolimus for 72 h and cell viability was determined as described in patients and methods. Results are expressed as mean ± SEM (n=3) in percent of control. Each assay was performed in triplicate. (**) p<0.01; (°°) p<0.01 versus control. **(B)** Cell viability determined as in A in primary culture cells from 22 pNETs incubated without (vehicle) and with 1 nM everolimus for 72 h. (NS = not significant). **(C)** Cell viability determined in B in the primary cultures of the 20 pNETs responsive to 1nM everolimus. The horizontal bar represents the median.

We first examined a dose effect of everolimus treatment for 72h ranging from 0.1nM to 10nM on cell viability of 3 pNETs primary cultures. Everolimus significantly reduced viable cell number at both 1 and 10nM with maximal effect observed for 1nM (Figure 1A). We then tested the effect of 1nM everolimus during 72h, in the primary cultures from 22 pNETs. This concentration is approximately the blood concentration of everolimus (range 2.7-95 nM) measured after 24h treatment of patients with 5-10mg per day [23, 24]. Everolimus decreased viable cell number from 14 % to 77 % of control value in 20 primary cultures (Figure 1B) with a median value of 59.3% (Figure 1C). Cell viability was not significantly altered in 2 primary cultures (90 ± 12% of control value; Figure 1B). No correlation was observed between cell viability inhibition by everolimus and the WHO grade or Ki67 index of the initial tumor (R=-0.207, p= 0.355; R=- 0.122, p=0.590 respectively). However, cell viability was repressed from 40 to 58% by everolimus in 4 out of 6 tested tumors with a Ki67≥10% (two grade 3 with Ki67≥ 45%). In the same way, no correlation was observed between cell viability inhibition by everolimus and the presence of *MEN1* tumor mutations or PTEN, DAXX/ATRX expression levels.

### Effects of everolimus on CgA secretion

**Figure 2:**
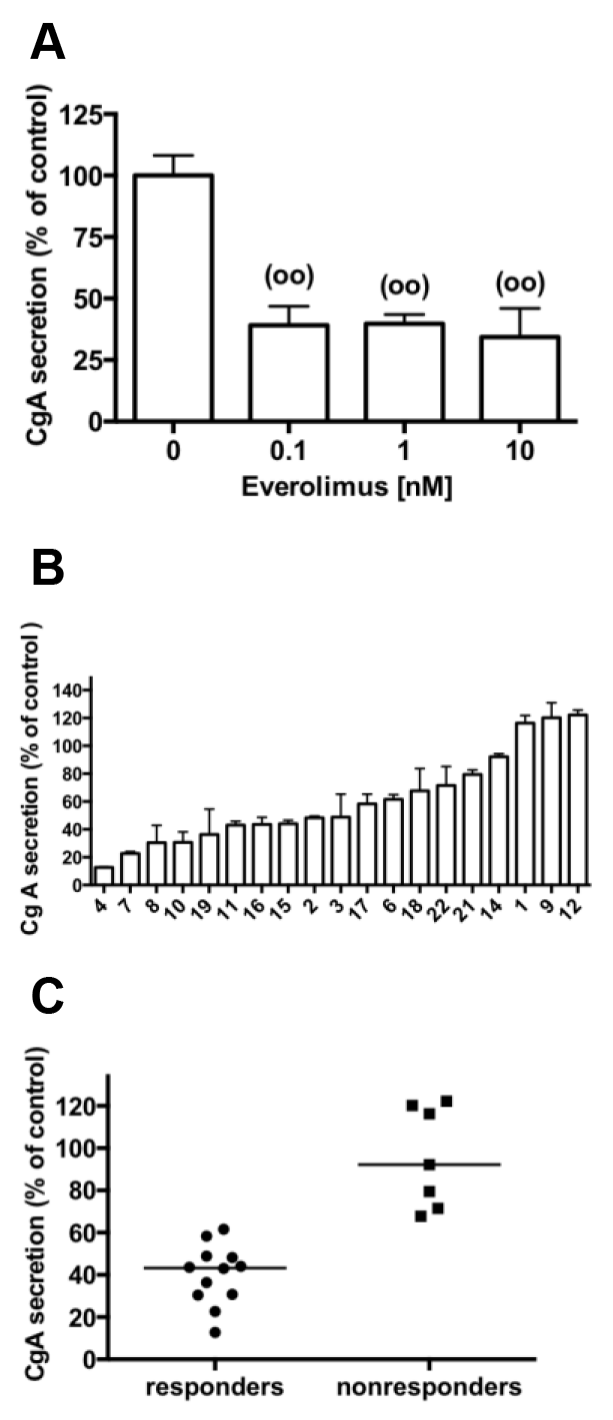
Effect of everolimus on CgA secretion in primary cultures of human pNETs **(A)** Primary culture cells from 3 pNETs (tumors 2,10,11) were incubated without (vehicle with 10^6^ fold DMSO dilution) and with 0.1-10 nM everolimus for 72 h and CgA secretion was measured as described in patients and methods. Results are expressed as mean ± SEM (n=3) in percent of control. Each assay was performed in triplicate. (°°) p<0.01 versus control. **(B)** CgA secretion determined as in A in primary culture cells from 19 pNETs incubated without (vehicle) and with 1 nM everolimus for 72 h. **(C)** CgA secretion determined in B of the 12 pNETs primary cultures that were responsive to 1nM everolimus treatment and CgA secretion of the 7 pNETs primary cultures that were nonresponsive to everolimus treatment. The horizontal bars represent the medians.

In the same 3 pNETs primary cultures described above, everolimus significantly decreased CgA secretion with the same efficacy regardless the concentration (Figure 2A). Among the 19 primary cultures analyzed, 1nM everolimus reduced CgA secretion from 13 % to 61 % of the control value in 12 primary cultures of pNETs with a median value of 43.3% (Figure 2B, 2C). CgA secretion was not significantly modified under 1nM everolimus treatment in the primary cultures from 7 tumors (median value = 92.2% of control) (Figure 2B, 2C). There was no correlation between everolimus inhibitions observed on cell viability and on CgA secretion (R=0.036, p=0.883). No correlation was observed between the inhibition of CgA secretion by everolimus and the WHO grade or Ki67 index of the initial tumor (R=0.237, p= 0.328; R=0.134, p=0.584 respectively). However, CgA secretion was repressed from 37 to 69% by everolimus in 4 out of 5 tested tumors with a Ki67≥10% (two grade 3 with Ki67≥ 45%). Moreover, no correlation was observed between CgA inhibition by everolimus and the presence of *MEN1* tumor mutations or PTEN, DAXX/ATRX expression levels.

### Effects of everolimus and SSAs in combination, on cell viability and caspase activities

**Figure 3:**
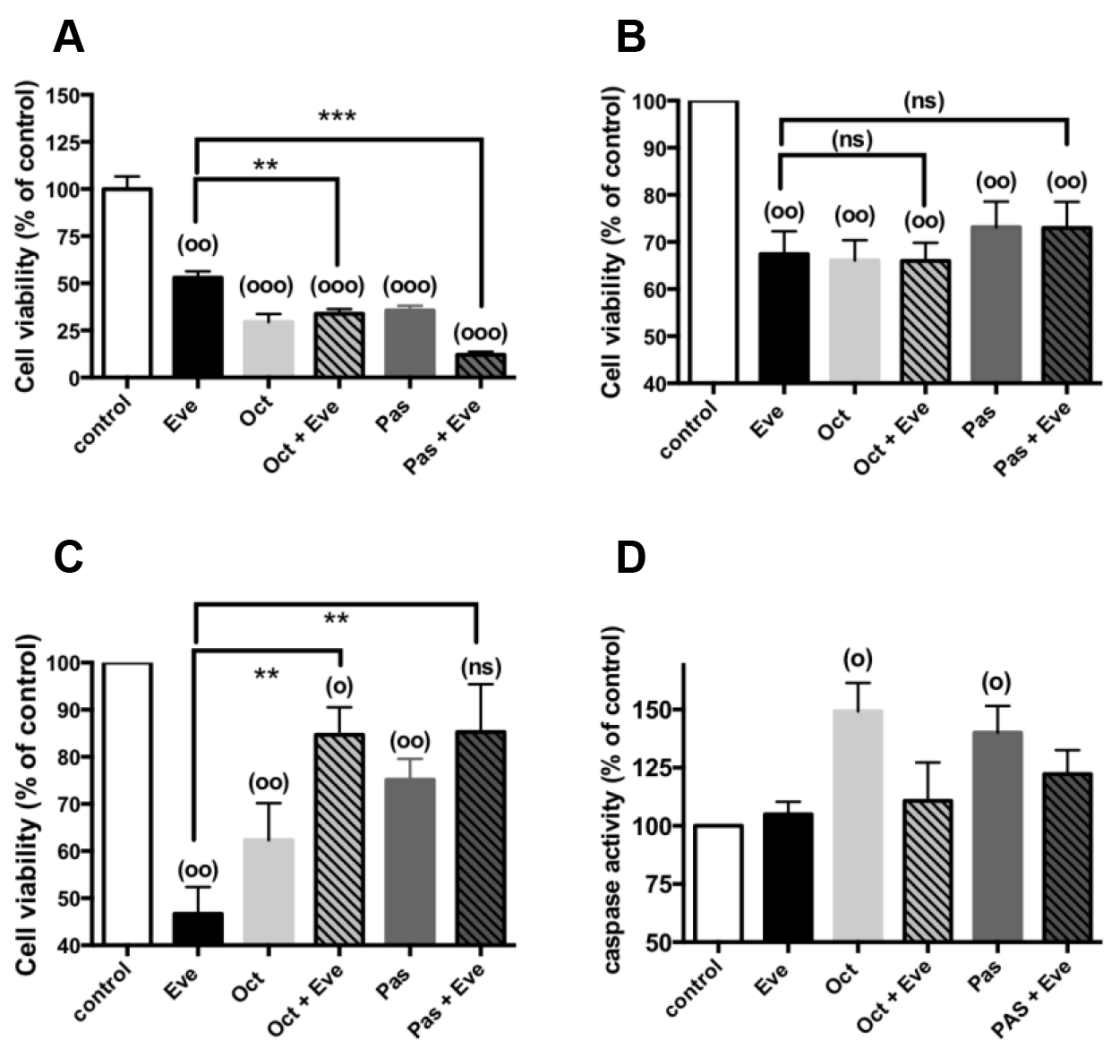
Cell viability and caspase activities in combined treatments of everolimus with SSAs Primary culture cells from **(A)** one pNET (n^°^8), **(B)** 9 pNETs (N^°^ 5,10-14,18-20) **(C)** 9 pNETs (n°1-4,6,7,9,15,17) were incubated for 72h in the absence (vehicle) or in the presence of 1nM everolimus (Eve), or 1nM octreotide (Oct), or 1nM pasireotide (Pas) alone (diluted in vehicle) or in the presence of everolimus and octreotide or everolimus and pasireotide as indicated in the figure. Cell viability was determined as described in patients and methods. Results are expressed as mean ± SEM (**(A)**, n=1; **(B,C)** n=9) in percent of control. **(D)** Primary cultures from 6 pNETs (n^°^1-3,7,9,18) were incubated for 24h in the absence (vehicle) or in the presence of 1nM everolimus and/or SSAs (diluted in vehicle) as indicated in the figure. Caspase activity was determined as described in patients and methods. Results are expressed as mean ± SEM (n=6) in percent of control. Each assay was performed in triplicate. (°) p<0.05; (°°) p<0.01 (°°°) p<0.001 versus control; (**) p<0.01; (***) p<0.001, (ns) not significant.

We have previously shown that, at clinically relevant concentrations [25, 26], both octreotide and pasireotide repressed hormonal secretion and cell viability in established pNETs primary cultures [20]. We then tested combined treatment of everolimus and SSAs, on cell viability in 19 pNETs responsive to everolimus in primary cultures. As previously described [20], 72h exposure to 1nM octreotide or 1nM pasireotide reduced cell viability with no significant difference between both analogs (Figure 3A, 3B, 3C). We have previously shown a strong correlation between the effects of octreotide and pasireotide on cell viability. A slight correlation was observed between everolimus and octreotide effects on cell viability whereas no correlation was observed between everolimus and pasireotide (R=0.492, p=0.044; R=0.217, p=0.402 respectively). No significant correlation was observed between the inhibition of cell viability by each SSA and the WHO grade or Ki67 index of the initial tumor (R< 0.07, p> 0.786 in the different cases). However, cell viability was largely repressed by octreotide, in 2 out of 5 tested tumors with a Ki67≥10% (by 53% in a grade 2 tumor with a Ki67=10% and by 74% in a grade 3 tumor with Ki67= 90%). We did not observed any correlation between SSAs-induced cell viability inhibition and the presence of *MEN1* tumor mutations or PTEN, DAXX/ATRX expression levels.

Cells from the different pNETs did not respond similarly to combined treatments of 1nM everolimus with either 1nM octreotide or 1nM pasireotide. Everolimus-induced inhibition of cell viability was increased in only one primary culture (from a grade 2 tumor with Ki67=3%; tumor n^°^8) under combined treatments (Figure 3A). Two types of response were observed for the other 18 primary cultures. Cell viability was significantly inhibited whatever the treatment in 9 primary cultures, without significant difference between the different treatments, even in combination (Figure 3B). These primary cultures were from 2 grade 1 and 7 grade 2 tumors, with Ki67≥5% in 6 tumors. Cell viability was also significantly inhibited by single treatments in the remaining 9 primary cultures with a significantly higher sensitivity to everolimus than SSAs (p=0.02 and p=0.004 respectively for octreotide and pasireotide). Surprisingly, combined treatments of 1nM everolimus with either 1nM octreotide or 1nM pasireotide, in these primary cultures, partially or fully reversed inhibition of cell viability induced by each single treatment (Figure 3C). Moreover, primary cultures of this group (Figure 3C) are significantly more responsive to everolimus than primary cultures responding similarly to each treatment (Figure 3B) (p< 0.02). These primary cultures were from 3 grade 1, 4 grade 2 and 2 grade 3 tumors with Ki67≥5% in 5 tumors. There was no significant difference between the two type of response to combined treatments with respect to the grade or Ki67 index of the initial tumor (p=0.999, p=0.746 respectively).

To elucidate the observed effects of everolimus alone or in combined treatment with the SSAs on cell viability, we determined the activation level of the executioner caspases 3/7. Basal caspase activities were not modified with 1nM everolimus treatment during 24h whereas both 1nM octreotide and 1nM pasireotide significantly increased caspase activities as previously observed associated to an increase in the number of TUNEL positive cells ([20], Figure 3D). Caspase activities were not significantly triggered by combined treatments of 1nM everolimus with either 1nM octreotide or with 1nM pasireotide (Figure 3D). This suggests that everolimus repressed the activity of caspases induced by SSAs in single treatments. Caspase activities have been determined in 6 primary cultures. Five of them belong to primary cultures described in Figure 3C and one in Figure 3B without difference in the response.

### Effects of everolimus and SSAs in combination on CgA secretion

**Figure 4:**
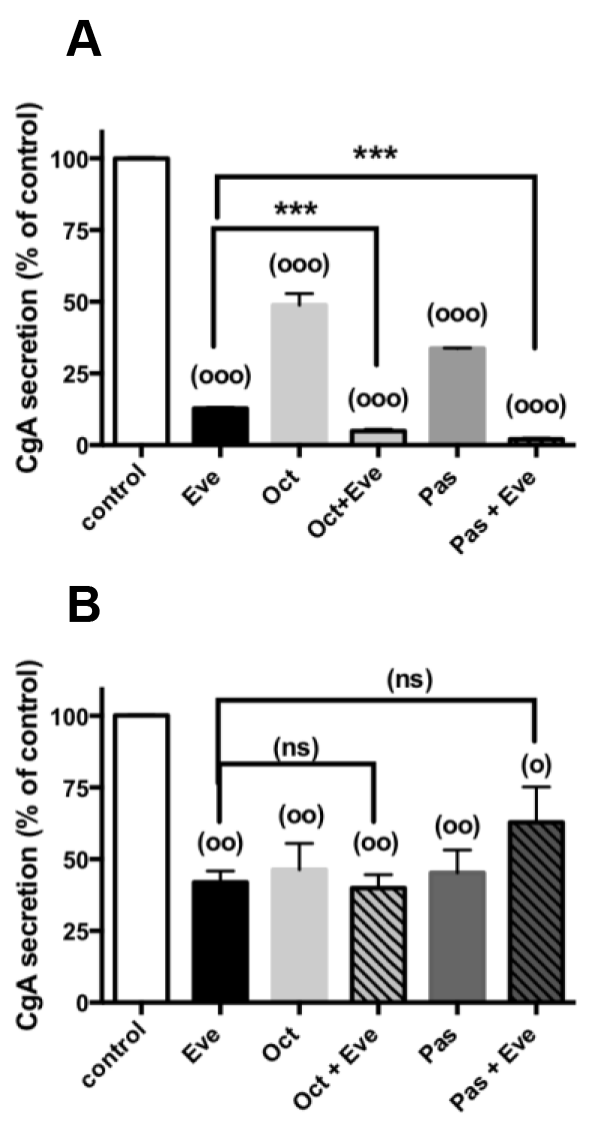
CgA secretion in combined treatments of everolimus with SSAs Primary culture cells from **(A)** one pNET (n^°^4), **(B)** 10 pNETs (n° 3,6-8,10,11,15-17,19) were incubated for 72h in the absence (vehicle) or in the presence of 1nM everolimus (Eve), or 1nM octreotide (Oct), or 1nM pasireotide (Pas) alone (diluted in vehicle) or in the presence of everolimus and octreotide or everolimus and pasireotide as indicated in the figure. CgA secretion was determined as described in patients and methods. Results are expressed as mean ± SEM (**(A)** n=1; **(B)** n=10) in percent of control. Each assay was performed in triplicate. (°) p<0.05; (°°) p<0.01 (°°°) p<0.001 versus control; (***) p<0.001, (ns) not significant.

We next explored the effect of everolimus combined with the SSAs on CgA secretion from the 12 pNETs responsive to everolimus in primary cultures. 72h treatment of cells with 1nM octreotide or 1nM pasireotide alone similarly decreased CgA secretion as previously observed [20] (Figure 4A, 4B), except in one primary culture which do not respond to SSAs (tumor 2; Table 2). We did not observe any correlation between everolimus and octreotide or everolimus and pasireotide inhibition of CgA secretion (R=0.205, p=0.522; R=0.342, p=0.276 respectively). Inhibition of CgA secretion by combined treatments was increased in only one primary culture (from a grade 1 tumor, with Ki67<2%; tumor 4) in comparison to everolimus or SSAs treatments alone (Figure 4A). We noticed that in this primary culture, combined treatments partially reverted cell viability inhibition induced by each single treatment. CgA secretion was significantly inhibited in the other 10 primary cultures whatever the treatment (Figure 4B). However no significant difference was observed between the different treatments, even in the combined treatments of 1nM everolimus with 1nM octreotide or 1nM pasireotide (Figure 4B). These 10 primary cultures were from 2 grade 1, 6 grade 2 and 2 grade 3 tumors with Ki67≥5% in 7 tumors. We did not observe any correlation between tumor grade, Ki67 index, *MEN1* tumor mutations or PTEN, DAXX/ATRX expression levels of the initial tumor and inhibition of CgA secretion by the different treatments (R< 0.156, p>0.109). These results suggested that although everolimus and SSAs reduced cell viability and CgA secretion in single treatments, treatment combinations did not achieve any beneficial impact except in two tumors (4, 8).

### Modulation of PI3K/Akt and ERK1/2 pathways under everolimus and SSAs single treatments

**Figure 5:**
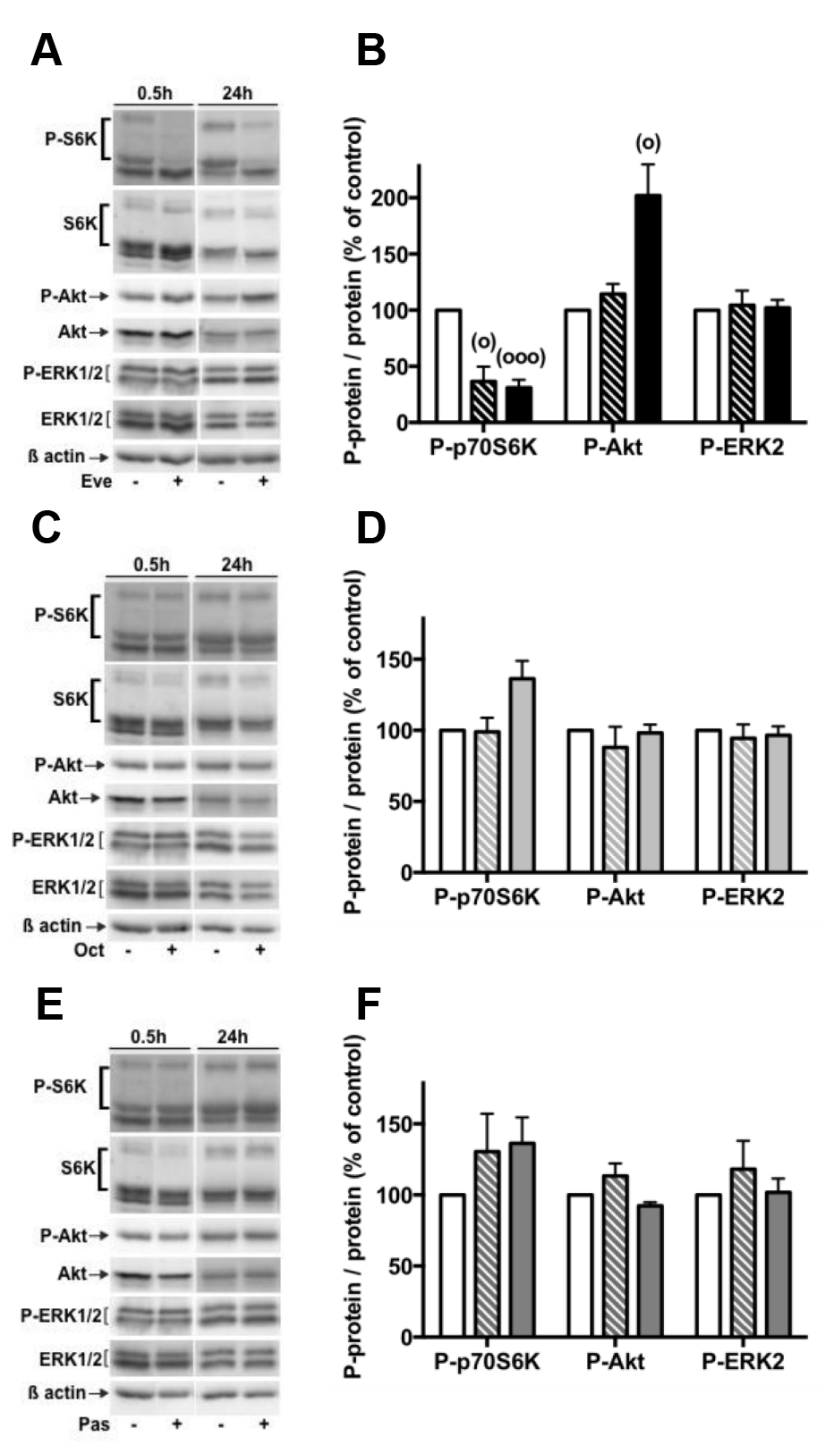
Effects of everolimus and the SSAs single treatments on the activity of the PI3K/Akt and MAPKinase ERK1/2 pathways Primary culture cells from pNETs were incubated 30 min (3 pNETs) or 24h in the absence ( vehicle) or in the presence of 1nM everolimus (Eve) (7 pNETs) **(A, B)** or 1nM octreotide (Oct) (5 pNETs) **(C, D)** or 1nM pasireotide (Pas) (5 pNETs) **(E, F)**. The phosphorylated levels of p70S6K, Akt and ERK1/2 were analyzed by using western blotting as described in patients and methods. Representative immunoblots are given in **(A)**, **(C)**, **(E)**. Means ± SEM in percent of control are given in **(B)**, **(D)**, **(F)** (**(B)** n=7; **(D,F)** n=5). Control condition (white bars), 30min treatment (hatched bars), 24h treatment (black and grey bars). (°) p<0.05, (°°°) p<0.001versus control.

It has been shown, in neuroendocrine cell lines or tumors, that everolimus and SSAs might control cell proliferation through the modulation of the PI3K/Akt/mTOR and/or the MAPKinase ERK1/2 pathways. In this context, we first explored the phosphorylated level of key intermediates of both pathways under everolimus and SSAs single treatments. We assessed the effect of everolimus, inhibitor of mTORC1, on its downstream target phosphorylation, p70S6K. After 30 min and 24h of treatment with 1nM everolimus, p70S6K phosphorylation was largely decreased, confirming that pNETs cells in primary cultures are sensitive to everolimus (Figure 5A, 5B). In these conditions, Akt phosphorylation was not modified during short treatment (30min), whereas it was increased after 24h of treatment (Figure 5A, 5B). Noted that although phospho-p70S6K was repressed, phospho-Akt (P-Akt) was not increased after 24h of everolimus treatment in two of the seven pNETs analyzed (tumors 5, 20). In addition, 1nM everolimus treatment did not alter ERK1/2 phosphorylation even after 24h of treatment (Figure 5A, 5B). Similarly, treatment of pNETs in primary cultures with 1nM octreotide or 1nM pasireotide during 30 min or 24h did not significantly modify the phosphorylation levels of p70S6K, Akt or ERK1/2 (Figure 5C–5F). These results suggested that the SSAs did not modulate the activity of PI3K/Akt and ERK1/2 pathways in our experimental conditions.

### Effects of everolimus and SSAs in combined treatments on PI3K/Akt and ERK1/2 pathways

**Figure 6:**
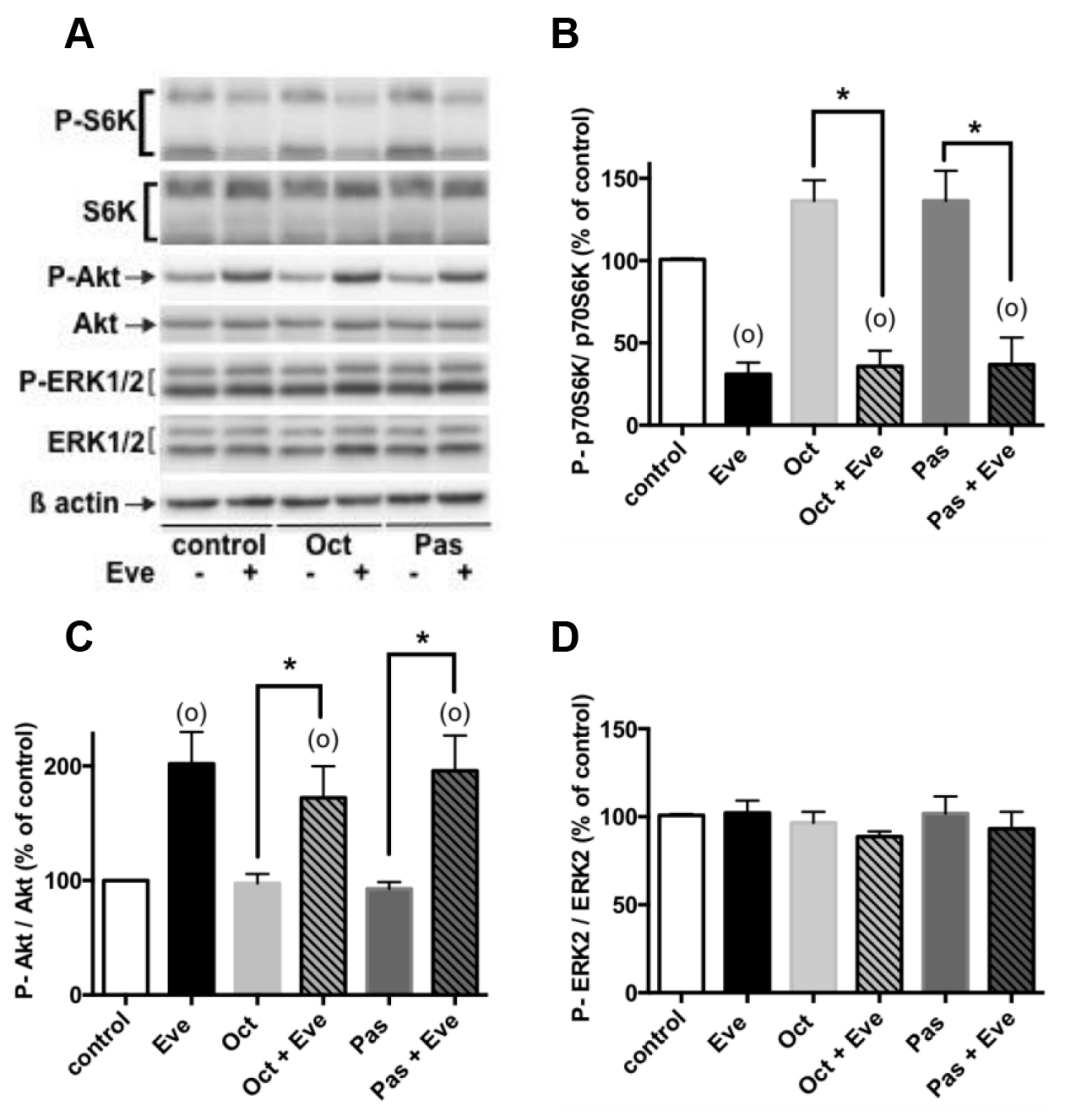
Effects of everolimus and the SSAs in combined treatments on the activity of the PI3K/Akt and MAPKinase ERK1/2 pathways Primary culture cells from 4 pNETs were incubated for 24h in the absence (vehicle) or in the presence of 1nM everolimus (Eve), or 1nM octreotide (Oct), or 1nM pasireotide (Pas) alone (diluted in vehicle) or in the presence of 1nM everolimus and 1nM octreotide or 1nM everolimus and 1nM pasireotide as indicated in the figure. The phosphorylated levels of p70S6K, Akt and ERK1/2 were analyzed by using western blotting as described in patients and methods. Representative immunoblots are given in **(A)**. Means ± SEM (n=4) in percent of control are given for p70S6K **(B)**, Akt **(C)** and ERK2 **(D)**. (*) p<0.05; (°) p<0.05 versus control.

We finally assessed the effects of combined everolimus and SSAs treatments on PI3K/Akt and ERK1/2 pathways. Combined treatments of 1nM everolimus and 1nM octreotide or 1nM pasireotide during 24h significantly inhibited p70S6K phosphorylation compared to control or SSAs in single treatment. However, SSAs did not enhance everolimus-induced inhibition of p70S6K phosphorylation (p=0.125; p=0.875 respectively in the presence of octreotide plus everolimus and pasireotide plus everolimus; Figure 6A, 6B). Likewise, combined treatments significantly increased P-Akt compared to control conditions or SSAs in single treatment (Figure 6A, 6C). However, P-Akt level was similar in both everolimus as single treatment or in combined treatments (p=0.796; p=0.750 respectively in the presence of octreotide plus everolimus and pasireotide plus everolimus). In the same conditions, drugs combination did not alter ERK1/2 phosphorylation (Figure 6A, 6D) as previously observed in the presence of each drug alone (Figure 5).

## DISCUSSION

Therapeutic strategy in the management of advanced pNETs is still challenging. Despite promising efficacy of targeted therapies observed in cell lines, PFS and overall survival of patients in clinical trials are lower than expected, suggesting lack of relevant preclinical models. We recently established and characterized primary cultures of human pNETs and demonstrated the anti-secretory and anti-proliferative effects of both octreotide and pasireotide [20]. In this report, we show for the first time that the mTOR inhibitor everolimus reduces both CgA secretion and cell viability in human primary cultures from pNETs. Although everolimus and SSAs decrease cell viability and CgA secretion in single treatments, no benefit is observed from combined treatments. To further delineate the mechanisms contributing to these effects, we demonstrate that everolimus-induced decrease of cell viability is not due to caspase-dependent apoptosis. Moreover, in combined treatments, caspase activities induced by SSAs are reverted. Considering two main signaling pathways previously shown as involved in NETs tumorigenesis [27], we show that ERK1/2 pathway is not affected by everolimus or SSAs as single or combined treatments in pNETs primary cultures. However, everolimus hyperactivates Akt, whereas SSAs do not alter PI3K/Akt/mTOR pathway. Moreover, in combined treatments, p70S6K activity is inhibited and Akt is as much activated as in the presence of everolimus alone.

The anti-proliferative effect of everolimus has been largely established in different NET cell lines [28–30] and particularly of pancreatic origin [13, 31, 32]. For the first time, we show that everolimus represses cell viability in the majority (90%) of primary cultures from human pNETs. The efficacy of everolimus is nevertheless variable and may correlate with the observations of the phase II and III clinical trials [14, 19] showing that partial or overall responses were rarely observed despite increase in PFS.

Beside the antitumoral management, control of hormone hypersecretion is required for up to 30% of patients with pNET. Advances in the control of hormone-excess state have been recently reviewed [33]. Whereas somatostatin analogs are the first-line treatment in the majority of GEP-NET patients, everolimus has been shown effective at controlling hormone-excess symptoms in patients with insulinoma in case reports or in small series [34–37] and in patients with carcinoid syndrome [38]. In clinical trials, CgA blood level is commonly considered as a biomarker of NETs. An early decrease in CgA blood level in response to everolimus has been correlated with an increase in PFS. So this early CgA response might have a global predictive value [19]. Here we show that everolimus represses CgA secretion in 63% of the pNETs primary cultures. Since decrease in cell number by everolimus treatment should have an impact on CgA secretion, the level of CgA secretion has been normalized to the cell number. Moreover, there is no correlation between everolimus-induced inhibition of cell viability and CgA secretion, suggesting that everolimus inhibition of CgA secretion might be a direct effect on pNET cell secretion. The PI3K pathway has been identified as a potential regulator of peptide secretion in the pNET cell lines BON and QGP1 depending on the inhibitor used (PIK-75, BKM120 or BEZ235) and the peptide secreted (neurotensin, serotonin or CgA) [39, 40]. Inhibition of both PI3K and mTOR seems to be required to block peptide secretion [40]. In the primary cultures of pNETs, inhibition of mTOR seems to be sufficient to repress CgA secretion (see below). Inhibition of CgA secretion under everolimus treatment is of importance as it has been previously shown that CgA and some of its fragments induce cell proliferation of the small intestinal NET cell lines L-STS and H-STS through Akt/mTOR activation [41].

Akt activation following rapalogs treatments is commonly observed in cancer cells. This effect has been attributed to loss of physiological negative feedback loops on PI3K/Akt and MAPK pathways, limiting the anti-proliferative potential of these drugs [15, 16, 18]. Activation of Akt by rapalogs is not systematically observed in NET cell lines [13, 30–32]. In the present report, all pNETs primary cultures tested are sensitive to everolimus (using p70S6K inhibition as downstream marker of mTOR inhibition). P-Akt (ser473) is increased whereas activation of ERK1/2 is never observed. We noticed that P-Akt is not upregulated in two tumors (5, 20). These observations indicate a specific upregulation of the PI3K/Akt pathway through loss of P70S6K/IRS-1 or P70S6K/mTORC2 negative feedbacks [15, 42]. In these conditions, everolimus does not induce caspase activity. This suggests that proapoptotic substrates downstream of both Akt and p70S6K, may be selectively phosphorylated and inactivated by Akt [43, 44]. Our results are in agreement with those of Valentino et al., showing an inverse correlation between Akt activation and apoptosis in BON and QGP1 cell lines [40]. Whereas everolimus induced both apoptosis and cell cycle arrest in cell lines [13, 45], its anti-proliferative effect occurs mainly through cell cycle arrest in the majority of NET cell lines [28, 30, 46]. This mechanism is likely predominant in primary cultures of pNETs and may contribute to disease stabilization observed in patients [14].

SSAs have a favorable safety profile in patients with NETs [6, 47]. Moreover, previous reports have shown that octreotide decreases PI3K/Akt pathway activity in pituitary and pancreatic adenocarcinoma cell lines [17, 18]. Since we have previously shown efficacy of SSAs in pNETs primary cultures [20], it was tempting to perform co-treatments of everolimus with SSAs to improve everolimus efficacy. Unfortunately, we cannot evidence any increase in either cell viability inhibition nor in the inhibition of CgA secretion in combined treatments (except in one pNET for each parameter analyzed). Similar observations have been made on primary cultures of human bronchial carcinoids treated with everolimus and pasireotide, although bronchial carcinoid cells are less responsive than pNETs cells to these agents as single treatments [48]. Lack of increase in the anti-proliferative effect of everolimus combined with octreotide has already been observed in the insulinoma rat cell line INS1, which is well sensitive to octreotide. In this cell line, both agents inhibit PI3K/Akt/mTOR pathway downstream of Akt through repression of TSC2, mTOR and p70S6K phosphorylation. Combined treatments did not enhance this effect, whereas Akt phosphorylation is not affected [31]. On the contrary, combined treatments of rapamycin and octreotide proved to be efficient in the pituitary cell line AtT20 and in human nonfunctioning pituitary adenoma cells in primary culture. In the AtT20 cells, octreotide increases IRS-1 phosphorylation that was suppressed by rapamycin, subsequently decreasing rapamycin-dependent Akt activation triggering cell cycle arrest through an increase in p27/kip1 level [30]. In human pNET cells octreotide and pasireotide do not repress the PI3K/Akt/mTOR pathway neither affect ERK1/2 pathway, although they decrease cell viability. Therefore, these pathways do not consistently mediate SSAs efficacy. In combined treatments with everolimus, SSAs are not able to repress everolimus-dependent Akt activation. Moreover, SSAs-dependent caspases activation is reverted, suggesting that persistent high level of P-Akt in combined treatments may counteract the cell death machinery elicited by SSAs by phosphorylation and inactivation of pro-apoptotic components [43]. Meric-Bernstam *et al*. recently showed that high level of P-Akt (detected in everolimus pre- and on-treatment tumor biopsies) correlated with longer PFS of patients carrying NET. They concluded that increase in P-Akt under everolimus treatment is not a marker of everolimus resistance but rather a marker of everolimus sensitivity [22]. In this clinical trial, all patients received octreotide LAR co-treatment [49], suggesting that, octreotide cannot repress everolimus-dependent Akt activation in the tumors of patients *in vivo*. These data are consistent with our results on primary cultures of pNETs.

Whereas decrease in objective response rate in combined treatments of everolimus and octreotide in clinical trial [19] may result from loss in caspase-dependent apoptosis observed in pNETs cells in primary culture, PFS is increased. This could be explained by indirect mechanisms involving everolimus and SSAs-induced inhibition of tumor angiogenesis that might contained tumor growth [50]. As a matter of fact, GEP-NETs are highly vascularized tumors expressing several growth factors and their corresponding receptors [51]. Among them, GEP-NETs synthesize and secrete high level of vascular endothelial growth factor (VEGF), a potent pro-angiogenic factor [52]. High expression of VEGF correlated with increased angiogenesis and decreased PFS [53]. Both SSAs [54, 55] and rapalogs [56] impaired VEGF production and secretion in tumors cells and particularly in NET cells [57, 58]. Moreover, they have been shown to control key steps of the angiogenic process: endothelial cell proliferation, endothelial tubule formation [50, 56, 59–61] which could be involved in the PFS increase observed in SSA and everolimus combined treatments in clinical trials. In a phase III trial, sunitinib a multi-tyrosine kinase receptors inhibitor targeting the VEGF receptor improved PFS as compared to placebo in patients with advanced pNETs [62] and obtained approval from the FDA.

In conclusion, this study represents the first investigation of combined treatments of everolimus with octreotide or pasireotide in primary cultures of human pNET cells. It shows that everolimus and SSAs repress cell viability and CgA secretion in single treatment, however their efficacy is not improved in combined treatments. These results are consistent with clinical reports showing that combined treatment do not induce a marked benefit for patients compared to everolimus single treatment. Akt activation by everolimus, unchallenged by SSAs, and loss of caspase-dependent apoptosis induced by SSAs are molecular events that strongly support this limited benefit. During the processing of our manuscript, Falletta *et al*. established a link between everolimus-dependent cell viability of pNET primary cultures, active Akt/mTOR/4EBP basal levels in the initial tumor tissue and the clinical aggressiveness of the tumor (high Ki67 index) [63]. In our study, we cannot highlight any correlation between cell viability or CgA secretion responses to everolimus and/or SSAs treatments and the WHO grade or Ki67 index of the initial tumor. Nevertheless, we observed good responses to everolimus or SSAs in some primary cultures from grade 3 tumors and/or Ki67≥10%. No correlation could be established between treatments responses and *MEN1* mutations, PTEN or DAXX/ATRX expression levels. This suggested that these available biomarkers are not predictive for the therapeutic response of pNETs to everolimus or SSAs [64]. Finally, primary culture of pNETs is a suitable preclinical model to further define biological effects and underlying mechanisms for new or other inhibitors of signaling pathways (Ras/raf/MEK, PI3K, IGF1R…) previously described as potentially promising for co-targeted therapy in NET cell lines [45, 46, 65–67].

## PATIENTS AND METHODS

### Patients

The present study was approved by the Ethics Committee of the Aix-Marseille University (Aix-Marseille, France) and informed consent was obtained from each patient. Twenty five patients with pNET tumor were included in the study (Table 3). All the patients were bearing tumor requiring surgical removal. Patients were naïve of antitumor treatment before surgery. The only criterion for tumors selection was their identification as primary pNET or pNET derived metastasis by pathological analysis. Twenty four tumors were primary pNETs and one was a hepatic metastasis of a pNET. In 22 cases, there was no clinical or biochemical evidence of hormonal hypersecretion, thus they were considered as nonfunctional pNETs. In 3 cases, the tumors were secreting and symptomatic: insulinoma (tumor 18), and gastrinoma (tumors 19,22), confirmed by pathology reports. Determination of Ki67 and proliferation index allowed grade classification (WHO 2010 classification) (Table 3).

**Table 3:** Clinical and pathological information of pNET patients

Tumor (number)	Age (year)	Sex	Tumor size (mm)	Liver metastasis	hormonal secretion	N	Ki67 (%)	mitosis	Grade	cured after surgery	treatment after surgery	post-surgical follow-up
1	67	F	44	No	non functioning	0/3N	1.5	3/10	G2	No	SSA started recently for appearance of liver metastasis 3 years after surgery	3 years
2	53	M	15	No	non functioning	NA	<2	<2/10	G1	Yes	No	1 year - lost
3	44	M	40	Yes	non functioning	5/8N	5	4/10	G2	No	No (stable liver metastasis)	3 years
4	66	F	55	No	non functioning	1/7N	<2	1/10	G1	Yes	No	3 years
5(a)	30	F	40	Yes	non functioning	Nx	10	1/10	G2	No	progression of liver metastasis, SSA started recently (somatuline)	2 years
6	67	M	25	No	non functioning	0/1N	90	10/10	G3	NA	NA	lost
7	75	F	15	No	non functioning	NA	<1	0/10	G1	Yes	No	6 month
8	59	F	35	No	non functioning	3/16N	3	NA	G2	Yes	No	4 years
9	76	M	48	No	non functioning	0/6N	5	2/10	G2	Yes	No	4 years
10	45	M	27	No	non functioning	0/6N	10	1/10	G2	Yes	No	1 year
11	49	F	45	No	non functioning	1/1N	13	15/10	G2	Yes	No	3 years
12	68	M	64	No	non functioning	0/15N	5	<1/10	G2	death after surgery	NA	
13	65	M	12	No	non functioning	NA	4	NA	G2	Yes	No	4 years
14	53	F	15	No	non functioning	NA	5	NA	G2	Yes	No	4 years
15	65	M	50	No	non functioning	5/27N	45	1/10	G3	Yes	No	3 years
16(b)	36	F	43(d)	No	non functioning	0/12N	5	1/10	G2	Yes	No	lost
17	66	F	50	No	non functioning	0/2N	6	<1/10	G2	No ( appearance of a liver metastasis)	chemotherapy (Adriamycin and streptozotocin)	4 years
18	48	M	80	Yes	Insulinoma	11/34N	10	NA	G2	No	SSA then Peptide receptor radionuclide therapy (7 cures)	3 years
19(c)	64	M	26(d)	No	gastrinoma	0/30N	<2%	<1/50	G1	No	PPI	5 years
20(a)	44	F	30	Yes	non functioning	6/19N	2	NA	G1	No	percutaneous thermoablation	2 years
21	69	M	55	No	non functioning	1/2N	<1	<1/10	G1	Yes	No	4 years
22	58	F	70	Yes	gastrinoma	2/5N	<2	NA	G1	NA	NA	lost
23	74	F	40	No	non functioning	5N/11N	22	NA	G3	Yes	no	1 year
24	30	M	75	No	non functioning	3/10N	40	24/10	G3	No (bone metastasis)	FOLFOX and radiotherapy on bone mestastasis	1 year-death
25	41	F	35	No	non functioning	0/3N	5	6/10	G2	Yes	No	1 year

Tumor size is expressed as maximal diameter (mm). For multiple localization, tumor size is the sum of each localization. (a) Tumor associated to MEN1 syndrome. (b) Tumor associated to VHL. (c) Patient treated with proton pump inhibitor (PPI) before surgery. (d) Multiple localization. NA (not available). N (lymph node). N: X/YN X positive nodes/Y analyzed nodes. Nx: no nodes were found in the resected specimen. Grade is determined according to WHO classification.

### Reagents

Everolimus, octreotide and pasireotide, were provided by Novartis (Novartis AG, Basel Switzerland). All other reagents were purchased from Sigma-Aldrich (St Quentin Fallavier, France). Stock solutions of 10^-2^M everolimus were prepared in DMSO and kept at -80°C. Serial dilutions to obtain final concentrations of 10nM to 0.1nM everolimus were done directly in the culture medium containing 5% Fetal calf serum (FCS) (corresponding final DMSO dilutions were from 10^6^ to 10^8^ fold). Stock solutions of octreotide (10^-3^M) and pasireotide (10^-2^M) were prepared into phosphate saline buffer containing 0.1% bovine serum albumin and 0.01N acetic acid and kept at -80°C. Serial dilutions to obtain 1nM octreotide or pasireotide were done in the culture medium containing 5% FCS. In combined treatments experiments, octreotide and pasireotide were diluted in the culture medium containing 5% FCS and DMSO (10^7^ fold dilution) when used as single treatment (diluted in vehicle). Culture medium containing 5% FCS and DMSO (10^7^ fold dilution) was used in control conditions (vehicle) in all the experiments containing everolimus. We have previously shown that 1nM octreotide or pasireotide were effective on pNET primary culture [20]. These concentrations are close to their blood plasma levels (range 2-6nM for octreotide and 4-15nM for pasireotide) in patients treated monthly with 30-120mg octreotide LAR or 20-60mg pasireotide LAR respectively [25, 26].

### Cell culture

Primary cultures of human pNETs were performed as previously described [20]. Tumor fragments of the 25 human pNETs, obtained after surgery, were dissociated mechanically and enzymatically [68]. Tumor cells were seeded into 24- or 6-well plates (according to the experiments) coated with extracellular matrix (ECM) (from bovine endothelial corneal cells as previously described [68]). Cells were maintained in culture in D-Valine DMEM, supplemented with 10% fetal calf serum (FCS), penicillin (100 U/ml), streptomycin (100 µg/ml), and glutamine (100 U/ml), at 37°C in a water-saturated atmosphere containing 7% CO2. Cell viability determination and chromogranin A (CgA) secretion were carried out on cell cultures from 22 and 20 tumors respectively. Other experiments were performed according to the quantity of available tumor cells after dissociation of each tumor (range: 1.5 10^6^ to 50 10^6^ cells). The number of primary cultures used for each experiment is indicated in the figure legends. All the experiments were done in D-Valine DMEM containing 5% FCS.

### Genomic analysis of *MEN1*

Formalin-fixed, paraffin-embedded tissue sections (FFPE) of 5µm from 25 pNETs were used for DNA extraction. One to three slides from each pNET were extracted with the QIAamp DNA FFPE Tissue Kit (Qiagen, Courtaboeuf, France) according to the size of tumor section. Briefly, xylene was applied on the slide and paraffin embedded tumor was scratched into a microfuge tube containing xylene. Extraction was then performed according to the manufacturers’ protocol. Purity and concentration of DNA were assessed using a Nanodrop ND-1000.

The coding exons and exon-intron boundaries of the *MEN1* gene (NM_003977.2) were PCR amplified and screened by direct sequencing using specific primers on an ABI 3500XLDx (Applied Biosystems). Genetic analysis was performed with variant reporter software and pathogenicity of allelic variations were evaluated *in silico* using a battery of different bioinformatics algorithms (Polyphen2, UMD-predictor and Alamut 2.2.0 software [69]. Only variants classified as probably pathogenic or pathogenic were considered (Table 1).

### Immunohistochemistry of PTEN, ATRX and DAXX

Immunohistochemical labeling was performed on 4µm unstained slide sections from Tissue microarray (TMA). Slides were deparaffinized and subjected to antigen retrieval. Immunolabelings for ATRX (rabbit Anti-ATRX antibody, dilution 1/400 (Sigma-Aldrich, Cat. # HPA001906)) and DAXX (rabbit Anti-DAXX antibody, dilution 1/100 (Sigma-Aldrich, Cat. # HPA008736)) were performed on the automated Ventana. Immunolabeling for PTEN (mouse Anti-PTEN antibody, dilution 1/50 (Dako, Les Ulis, France, Cat. # 6H2.1)) were performed on the automated Dako. The expression was scored using stromal cells as a positive internal control. For ATRX and DAXX, positive case was defined as nuclear staining within tumor cells and negative staining was defined as lacked nuclear immunolabeling. For PTEN, only cytoplasmic staining was studied: low cytoplasmic staining was defined as 1 and moderate to intense cytoplasmic immunolabeling as 2 and 3 respectively.

### Real-time quantitative PCR

Total mRNAs were extracted from 3×10^5^ cells using the RNeasy Microkit (Qiagen). SST2 receptor mRNAs were detected by real-time quantitative PCR using specific primers and probes as previously described [70]. To produce standard curve, cDNA construct was produced, verified by sequencing and linearized. The mRNA level of SST2 was normalized to the glucuronidase β (GUSβ) mRNA level, as previously described [70]. Results were expressed as copy of mRNA for the SST2 gene/copy of mRNA for GUSβ.

### Cell viability

Cells from each tumor were seeded into 24-well plates (4×10^4^ cells/well). After 24 h in culture, cells were treated or not with the different pharmacological agents, as indicated in the figure legends, for 72 h. The medium was recovered for CgA determination and cell viability was assayed by a luminescent assay (CellTiter Glo; Promega, Charbonnières les Bains, France) according to the manufacturer's protocol. Results were expressed as percentages of the value for control cells (in the absence of treatment).

### CgA secretion

CgA secretion was determined on the medium recovered from cell viability experiments after 72 h of treatment (see above). The recovered culture media were centrifuged at 400g and stored at -20°C. CgA was measured using an Elisa kit (Chromogranin A Elisa Kit, Dako) according to the manufacturer's protocol. CgA values were normalized to the corresponding cell viability values. Results were expressed as percentages of the value for the control conditions.

### Caspase activity determination

Cells from each tumor were seeded into 24-well plates (4×10^4^ cells/well). After 24 h in culture, cells were treated with the different pharmacological agents for 24 h. The activity of the executioner caspases 3 and 7 was measured using Caspase Glo 3/7 Assay (Promega) according to the manufacturer's protocol. Results were expressed as a percentage of the value for the control conditions.

### Protein extraction and western blotting

Cells from each tumor were seeded into 6-well plates (0.5×10^6^ cells/well). After 24 h in culture, cells were treated or not with the different pharmacological agents, as indicated in the figure legends. Cells were then solubilized at 4°C for 15 min in a lysis buffer [25mM Tris (pH7.4), 150 mM NaCl, 1% Nonidet P-40, 0.25% deoxycholate, 1 mM EGTA, 1mM 4-(2-aminoethyl)benzenesulfonyl fluoride (AEBSF), 1 mM Na_3_VO_4_, and 10 μg/ml leupeptin and aprotinin] as previously described [27]. Denatured proteins were separated on 10% SDS-PAGE and transferred to PVDF membrane (Perkin Elmer, France). Immunodetection of the β actin and the phosphorylated levels of Akt, ERK1/2 and p70S6K was performed using the mouse monoclonal anti-β-actin (Sigma-Aldrich, Cat. # A5441), the polyclonal phospho-AktSer473 (Cat. # 9271), phospho-p44/42ERK1/2 (Cat. # 9101), the monoclonal phospho-p70S6KThr389 (Cat. # 9234) rabbit antibodies respectively (New England Biolab, Ozyme, France) and an anti-mouse IgG or anti-Rabbit IgG coupled to alkaline phosphatase as the secondary antibody. Blots were developed with the enhanced chemiluminescence CDP-Star™ detection system (Life Technologies, France) and quantified using Syngene and Genetools sofwares (Gbox, Ozyme, France). The total Akt, ERK1/2 and p70S6K content was systematically monitored by reprobing the membrane using the polyclonal Akt (Cat. # 9272), the monoclonal p70S6K (Cat. # 2708) rabbit antibodies (New England Biolab, Ozyme, France) and the polyclonal ERK1 antiserum (Santa Cruz Biotechnology, Tebu, France, Cat. # sc-94). The relative expression of phospho-Akt, phospho-ERK2 and phospho-p70S6K was calculated as a ratio to each total protein respectively and expressed in percent of the control value.

### Statistical analysis

Each assay was performed in triplicate except for immunoblotting determinations. Results are presented as the mean ± SEM of at least three tumors. The statistical significance between groups was determined by the paired Wilcoxon nonparametric test or Student's t-test and unpaired Mann-Whitney test. To measure the strength of association between pairs of variables without specifying dependency, Spearman rank order correlations were run. To measure the link between two qualitative parameters, Chi square test was performed. Differences were considered statistically significant at p ≤ 0.05.
